# Draft genome sequences of *Phytophthora kernoviae* and *Phytophthora ramorum* lineage EU2 from Scotland

**DOI:** 10.1016/j.gdata.2015.09.010

**Published:** 2015-09-22

**Authors:** Christine Sambles, Alexandra Schlenzig, Paul O'Neill, Murray Grant, David J. Studholme

**Affiliations:** a Biosciences, University of Exeter, Devon, UK; b Science and Advice for Scottish Agriculture (SASA), Plant Biosecurity and Inspections, Edinburgh, UK

## Abstract

Newly discovered *Phytophthora* species include invasive pathogens that threaten trees and shrubs. We present draft genome assemblies for three isolates of *Phytophthora kernoviae* and one isolate of the EU2 lineage of *Phytophthora ramorum*, collected from outbreak sites in Scotland.

**Table:** 

Specifications	

Organism/cell line/tissue	*Phytophthora ramorum* and *Phytophthora kernoviae*
Sex	Not applicable
Sequencer or array type	Illumina HiSeq
Data format	Analyzed; i.e. raw data filtered and assembled.
Experimental factors	Genomic sequence of pure microbial cultures
Experimental features	Genomic sequence of pure microbial cultures
Consent	Not applicable. Data are available without restriction.
Sample source location	Scotland, United Kingdom

## 1 Direct links to deposited data

http://www.ncbi.nlm.nih.gov/bioproject?LinkName=assembly_bioproject&from_uid=529658.

http://www.ncbi.nlm.nih.gov/bioproject?LinkName=assembly_bioproject&from_uid=529668.

http://www.ncbi.nlm.nih.gov/bioproject?LinkName=assembly_bioproject&from_uid=529678.

http://www.ncbi.nlm.nih.gov/bioproject?LinkName=assembly_bioproject&from_uid=534508.

## 2 Experimental design, materials and methods

Newly discovered *Phytophthora* species are increasingly reported as invasive pathogens, threatening trees and shrubs in the natural environment as well as in public and heritage gardens. For example, *Phytophthora kernoviae* is a recently described species first isolated in the south west of England [1] from bleeding stem lesions on mature beech trees (*Fagus sylvatica*) and foliar and stem necroses on *Rhododendron ponticum*. It also infects a range of other woodland and ornamental trees and shrubs such as *Quercus robur*, *Quercus ilex*, *Magnolia* spp., *Pieris* spp. and heathland plants (e.g*. Vaccinium myrtillus*).

In the USA, *Phytophthora ramorum* is the causative agent of sudden oak death, killing millions of trees along the Pacific West Coast since the mid 1990s [2]. In Europe *P. ramorum* was initially associated with *Rhododendron* and *Viburnum* within the ornamental nursery trade but the known host range has now expanded to well over 100 species of trees, shrubs and herbaceous plants. In the UK, initial findings were quickly followed by spread into landscaped and wider environments driven mainly by *Rhododendron ponticum* and again affecting *Vaccinium myrtillus* in heathland. Few trees were infected in the UK until 2009, when *P. ramorum* began to rapidly spread through Japanese larch (*Larix kaempferi*) and to a lesser extend to other larch plantations, necessitating the premature felling of large numbers of trees [3]. Four distinct genetic lineages of *P. ramorum* are known: The NA1 and NA2 lineages are present in North America; in Europe are the EU1 lineage and the recently discovered EU2 lineage, which is currently restricted to Northern Ireland and the south-west of Scotland [4], [5]. Only the NA1 lineage has a previously published genome sequence [6]. We used the Illumina HiSeq to sequence genomic DNA of three isolates of *P. kernoviae* and one isolate of the EU2 lineage of *P. ramorum*, all isolated from different outbreak sites in Scotland (Table 1). Paired 100-bp reads were assembled *de novo* and scaffolded using Velvet v. 1.2.03 [7]. The availability of the first genome sequences for *P. kernoviae* and its comparison with other *Phytophthora* will facilitate insights into the infection biology of this invasive pathogen and identification of core sets of genes shared across the genus. Availability of the first genome sequence from the EU2 lineage will be a useful resource for investigating the relationships among the four lineages as well as developing assays for detection and monitoring.

Sequence data are available via the accession numbers listed in Table 1 and annotation of the genome assemblies is available via Ensembl Protists [8].

## Figures and Tables

**Table 1:** Sequenced isolates.

Isolate	Accession numbers: GenBank^1^ (and SRA)	Scaffold assembly size (bp)	Number of scaffolds	Scaffold N_50_ (bp)	Host	Date of isolation
*P. kernoviae* 00238/432	AOFI00000000 (SRX212407)	43,208,681	1805	72,999	*Rhododendron ponticum*	August 2010
*P. kernoviae* 00629/1	AOFJ00000000 (SRX212404)	43,295,191	2542	58,074	*Rhododendron ponticum*	May 2011
*Phytophthora kernoviae* 00844/4	AOFK00000000 (SRX212403)	42,716,609	2538	58,795	*Rhododendron ponticum*	October 2011
*P. ramorum* EU2 996/3	AOBL00000000 (SRX212402)	49,703,133	3450	51,489	*Magnolia stellata*	November 2011

1 Data have been deposited at GenBank under these accession numbers, and the version described in this paper is version XXXX01000000.
